# Analysis of global gene expression using RNA-sequencing reveals novel mechanism of Yanghe Pingchuan decoction in the treatment of asthma

**DOI:** 10.1186/s12890-024-02952-8

**Published:** 2024-03-18

**Authors:** Lingyu Pan, Bangfu He, Chunxia Gong, Yehong Sun, Xianchun Duan, Yanquan Han, Jijun Chu, Yongzhong Wang

**Affiliations:** 1 The First Affiliated Hospital of Anhui University of Chinese Medicine, 117 Meishan Road, Shushan District, 230031 Hefei, Anhui China; 2 Anhui University of Chinese Medicine, 230012 Hefei, Anhui China

**Keywords:** Yanghe Pingchuan decoction, Asthma, RNA-sequencing, Ang-II/PLA/CaM signaling axis, Airway smooth muscle

## Abstract

**Background:**

Yanghe Pingchuan decoction (YPD) has been used for asthma treatment for many years in China. We sought to understand the mechanism of YPD, and find more potential targets for YPD-based treatment of asthma.

**Methods:**

An ovalbumin-induced asthma model in rats was created. Staining (hematoxylin and eosin, Masson) was used to evaluate the treatment effect of YPD. RNA-sequencing was carried out to analyze global gene expression, and differentially expressed genes (DEGs) were identified. Analysis of the functional enrichment of genes was done using the Gene Ontology database (GO). Analysis of signaling-pathway enrichment of genes was done using the Kyoto Encyclopedia of Genes and Genomes (KEGG) database. Real-time reverse transcription-quantitative polymerase chain reaction was undertaken to measure expression of DEGs.

**Results:**

Pathology showed that YPD had an improvement effect on rats with asthma. RNA-sequencing showed that YPD led to upregulated and downregulated expression of many genes. The YPD-based control of asthma pathogenesis may be related to calcium ion (Ca2+) binding, inorganic cation transmembrane transporter activity, microtubule motor activity, and control of canonical signaling (e.g., peroxisome proliferator-activated receptor, calcium, cyclic adenosine monophosphate). Enrichment analyses suggested that asthma pathogenesis may be related to Ca2 + binding and contraction of vascular smooth muscle. A validation experiment showed that YPD could reduce the Ca2 + concentration by inhibiting the Angiopoietin-II (Ang-II)/Phospholipase (PLA)/calmodulin (CaM0 signaling axis.

**Conclusion:**

Control of asthma pathogenesis by YPD may be related to inhibition of the Ang-II/PLA/CaM signaling axis, reduction of the Ca^2+^ concentration, and relaxation of airway smooth muscle (ASM).

## Introduction

Bronchial asthma is also referred to as ‘asthma’. Asthma is a chronic inflammatory disease of the airways caused by the participation of various inflammatory cells, inflammatory mediators, and neurotransmitters [1]. Asthma is a common respiratory disease with a high incidence. According to a report from the Global Initiative for Asthma Committee, among more than 300 million patients suffering from asthma worldwide, more than 30 million patients reside in China [2]. In China, the incidence of asthma in young children is high [3]. Asthma is one of the most common chronic diseases in the world, and its incidence is increasing worldwide, bringing a high economic burden to society [4].

The etiology of asthma is incompletely understood. Clinical studies have shown genetic factors and the environment to be the main risk factors for inducing asthma [5, 6]. The pathogenesis of asthma is relatively complex and not fully elucidated. Research on the pathogenesis of asthma has focused mainly on chronic inflammation of the airways, airway hyperresponsiveness [7], airway remodeling [8], and genetic mechanisms [9].

The main focus of treatment for asthma is symptomatic relief and control of further development of the disease [10]. The main drugs used for asthma treatment are glucocorticoids [11], leukotriene-receptor antagonists [12], and β_2_-receptor agonists [13]. However, long-term use of these agents may elicit adverse reactions [14]. For example, prolonged use of high-dose glucocorticoids increases the risk of bacterial infections [15], eye, pharyngeal, and other diseases in children, as can affect the growth and development of children.

Traditional Chinese medicine (TCM) has unique characteristics in the treatment of asthma. The therapy of asthma based on TCM concepts centers on relieving symptoms during an attack and treating its cause after remission. Yanghe Pingchuan decoction (YPD) is a TCM formulation and has been used to treat asthma. YPD is composed of *Ephedra sinica* Stapf (mahuang), *Rehmannia glutinosa* (Gaertn.) DC (shudihuang), *Inula japonica* Thunb (xuanfuhua), *Morinda officinalis* F.C.How (bajitian), *Schisandra chinensis* (Turcz.) Baill (wuweizi), *Sinapis alba* L (baijiezi), *Draba nemorosa* L (tinglizi), *Angelica sinensis* (Oliv.) Diels (danggui), *Platycodon grandiflorum* (Jacq.) A.DC (jiegeng). YPD have also been used in the clinical treatment of asthma for many years with clear curative effects [16]. Previously, we demonstrated that YPD has a therapeutic effect on rats with asthma, and that its mechanism of action may be related to inhibition of the phosphoinositide 3-kinase/protein kinase B signaling pathway [17]. However, the specific molecular mechanisms underlying the effects of YPD are incompletely understood.

Herein, we attempted to elucidate the potential molecular mechanisms underlying the effects of YPD on a rat model of asthma using RNA-sequencing.

## Materials and methods

### Chemical reagent

Ovalbumin (Lot: CLCB9757) was purchased from Sigma-Aldrich (St. Louis, MO, USA). Calcium ion test kits were purchased from Jiancheng Bioengineering Research Institute (Nanjing, China). The RNA extraction kit were provided by Sangong Bioengineering (Shanghai Co., Ltd. China). Reverse Transcription Kit(with dsDNase) were provided by Biosharp (Hefei, China).

### YPD preparation

All of herbs of YPD were obtained from Anqing Huashi Chinese Herbal Medicine Beverage (Anqing City, China). YPD preparation involved three main steps. First, each herb was immersed in water (10×) for 1 h, and decocted by boiling for 2 h. Second, after filtration, the residue was decocted with water (8×) for 1.5 h. Third, the two decoctions were combined and concentrated to 0.2 g/mL in a vacuum at 50 °C to obtain a YPD suspension.

### Animal experiments and sample collection

The protocol for all animal experiments was approved (AHUCM-rats-2,022,093) by the Animal Experiment Ethics Committee of Anhui University of Chinese Medicine (Anhui, China).

All experimental methods and drug regimens were based on our previous work [17]. Male Sprague–Dawley rats (200 ± 20 g) were obtained from the Experimental Animal Center of Anhui Medical University. Rats were divided randomly into three groups of 10: control, asthma, and YPD. On day 1, rats in the asthma group and YPD group were injected (i.p.) with 10% ovalbumin and 1 mL of physiologic (0.9%) saline. On day 15, rats were placed in a box for nebulization (1% ovalbumin plus 0.9% saline, 400 mmHg, 15 min), once every 2 days for 10 days to create an asthma model. From day 28 of modeling, the YPD group was given YPD (14.76 g/kg, i.g.) and the control group and asthma group were given an identical amount of 0.9% saline, once a day, for 14 consecutive days. After the experiment, each rat was fasted for 8 h, and anesthetized with sodium pentobarbital [18, 19]. Blood was collected from the abdominal aorta, and samples were taken for testing.

### Histopathology

After killing, right lungs were harvested, fixed, embedded in paraffin, and sectioned. Changes in the structure of airway walls and pathologic alterations in bronchi and smooth muscles were observed by staining (hematoxylin an eosin) and analyzed using Image J (US Institutes of Health, Bethesda, MD, USA).

### Staining

Tissues were fixed in 4% paraformaldehyde at 55℃ for 1 h. The extent of bronchial fibrosis was determined by staining with Masson’s trichrome (1%) for 5–10 min at 55℃. Then, we evaluated the extent of fibrosis in lungs and bronchial tissue with an optical microscope and undertook pathology grading.

### RNA extraction

Total RNA was extracted and purified by the mirVana™ miRNA Isolation Kit (catalog number: AM1561; Ambion, Austin, TX, USA) according to manufacturer instructions. RNA quality was evaluated by a spectrophotometer (Nanodrop™; Thermo Fisher Scientific, Waltham, MA, USA) and a bioanalyzer (2100 series; Agilent Technologies, Santa Clara, CA, USA).

### Library preparation for transcriptome sequencing (RNA-sequencing)

RNA libraries were prepared using the SureSelect Chain-specific RNA Library Preparation kit (Agilent Technologies). Thereafter, a fluorometer (Qubit 3.0; Thermo Fisher Scientific) and bioanalyzer (2100 series; Agilent Technologies) were used to quantify the purified libraries. Cluster generation was undertaken using cBot (Illumina, San Diego, CA, USA). Sequencing was achieved with NovaSeq™ 6000 (Illumina). These procedures were outsourced to Hefei Yuan En Biotechnology Co., LTD (Hefei, China).

### Differentially expressed genes (DEGs)

FastQC (https://www.bioinformatics.babraham.ac.uk/projects/fastqc/) [15] was used for the quality control of experimental samples to ensure that reads of low quality, high N content of unknown bases, or adapter contamination were filtered out. Hisat (http://daehwankimlab.github.io/hisat2/) [15] was employed to count the reads numbers mapped to genes. To make the expression of different genes and different samples comparable, based on the length of the gene itself and the amount of sequencing data, we converted the reads into fragments per kilobase of exon model per million mapped reads (FPKM). EdgeR (https://bioconductor.org/) was used to analyze DEGs between samples. After obtaining the p-value, correction of the multiple hypothesis test was undertaken. The threshold of the p-value was determined by controlling the false discovery rate (FDR), and the corrected p-value was the Q value. Simultaneously, we calculated the fold change (FC) based on the FPKM value. The conditions for DEG screening were Q ≤ 0.05 and FC ≥ 2.

### Enrichment analyses

To understand more deeply the functions of DEGs, R (R Institute for Statistical Computing, Vienna, Austria) was used to analyze the enrichment of function based on the Gene Ontology (GO) database (http://geneontology.org/) [15] and enrichment of signaling pathways using the Kyoto Encyclopedia of Genes and Genomes (KEGG) database (www.genome.jp/) [15]. We used clusterProfiler (https://bioconductor.org/) to analyze enrichment of function and signaling pathways. The top-30 enriched functions and signaling pathways were obtained. The Search Tool for the Retrieval of Interacting Genes/Proteins (STRING) database (https://string-db.org/) [15] was employed to analyze protein–protein interaction (PPI) networks.

### Validation experiment

Real-time reverse transcription-quantitative polymerase chain reaction (RT-qPCR) was used to verify the reliability of RNA-sequencing results. Transcriptome analysis showed the genes to have upregulated expression were Ang-II, PLA and CaM, so they were selected for validation by real-time RT-qPCR. β-actin was an internal reference gene. The 2^−ΔΔ^ CT method was used to calculate the relative expression of genes.

RT-qPCR was applied to measure mRNA expression of Ang-II, PLA and CaM. Samples of total RNA were taken from right-lung tissues, seeded in a six-well culture plate at 1 × 10^6^ cells/well, allowed to incubate for 24 h, and exposed to lysis buffer (Science Biotech, Beijing, China). Then, the absorbance at 260 and 280 nm of total RNA was determined by an ultraviolet spectrophotometer to determine the mRNA concentration. mRNA was reverse-transcribed into complementary-DNA with a cDNA reverse transcription kit according to manufacturer instructions. RT-qPCR was done on the LightCycler™ 480 II system (Roche Diagnostics, Basel, Germany/) using 2 × S6 Universal SYBR qPCR Mix (EnzyArtisan, Shanghai, China). The thermal cycling conditions were pre-denaturation at 95 °C for 30 s, denaturation at 95 °C for 10 s, annealing and extension at 60 °C for 30 s for 45 cycles, and extension at 72 °C for 5 min. The genes targeted for amplification were Ang-II, PLA and CaM, with β-actin as the internal reference gene. The 2^−ΔΔCt^ method was applied to normalize the relative expression of mRNA. The primer sequences were devised and synthesized by EnzyArtisan, and are shown in Table 1.

**Table 1:** Sequences of the primers used in this study

Gene	Amplicon Size (bp)	Forward (5’→3’)	Reverse (5’→3’)
β-actin	150	CCCATCTATGAGGGTTACGC	TTTAATGTCACGCACGATTTC
CaM	89	AAGTGTGGAGTTGTGAGCGT	GAGTACCGGACAGAACACCC
Ang-II	118	GTACAGAGTAGCCTGGGAATAGAT	TGCTTCTGTGTGTCCTTTAGC
PLA	94	GAATCTGGAGCCCATGCACAC	TCCTGACTCCACTTGACCAAA

### Ca^2+^ was determined by methyl thymol blue (MTB) microplate method

We weighed tissue accurately. Nine times the volume of deionized water was added to tissue. The tissue homogenate was centrifuged (2500 × g, 10 min, room temperature). Then, 10% of the supernatant of the homogenate was removed for measurement. After cell samples had been collected, deionized water (0.3–0.5 mL) was added to 1 × 10^6^ cells. After ultrasonic crushing, samples were mixed, centrifuged (2500 rpm, 10 min, room temperature), and the supernatant removed for measurement. A 96-well plate that had been treated with dilute hydrochloric acid and cleaned with deionized water was selected. Blank holes, standard holes, and measurement holes were assigned (Table 2) and solutions added in sequence.

**Table 2:** Hole types used for validation experiment

	Blank hole	Standard hole	Measuring hole
Deionized water	10 µL		
1mmol/L Calcium standard solution (1 mmol/L)		10 µL	
Tissue-homogenate supernatant			10 µL
Working fluid II	250 µL	250 µL	250 µL
After mixing well and leaving for 5 min, the absorbance of each hole was measured by colorimetry at 610 nm			

### Statistical analyses

Results were analyzed by SPSS 23.0 (IBM, Armonk, NY, USA). Data are the mean ± SD. One-way ANOVA was used to test for significant between-group differences. Differences were considered significant at p < 0.05 or p < 0.01. Differential analyses of GO and KEGG pathway enrichment were analyzed and results were considered statistically significant at p < 0.05. The conditions for DEG screening were p ≤ 0.05 and|log FC| ≥ 2.

## Results

### Therapeutic effect of YPD on rats suffering from asthma

**Fig. 1:**
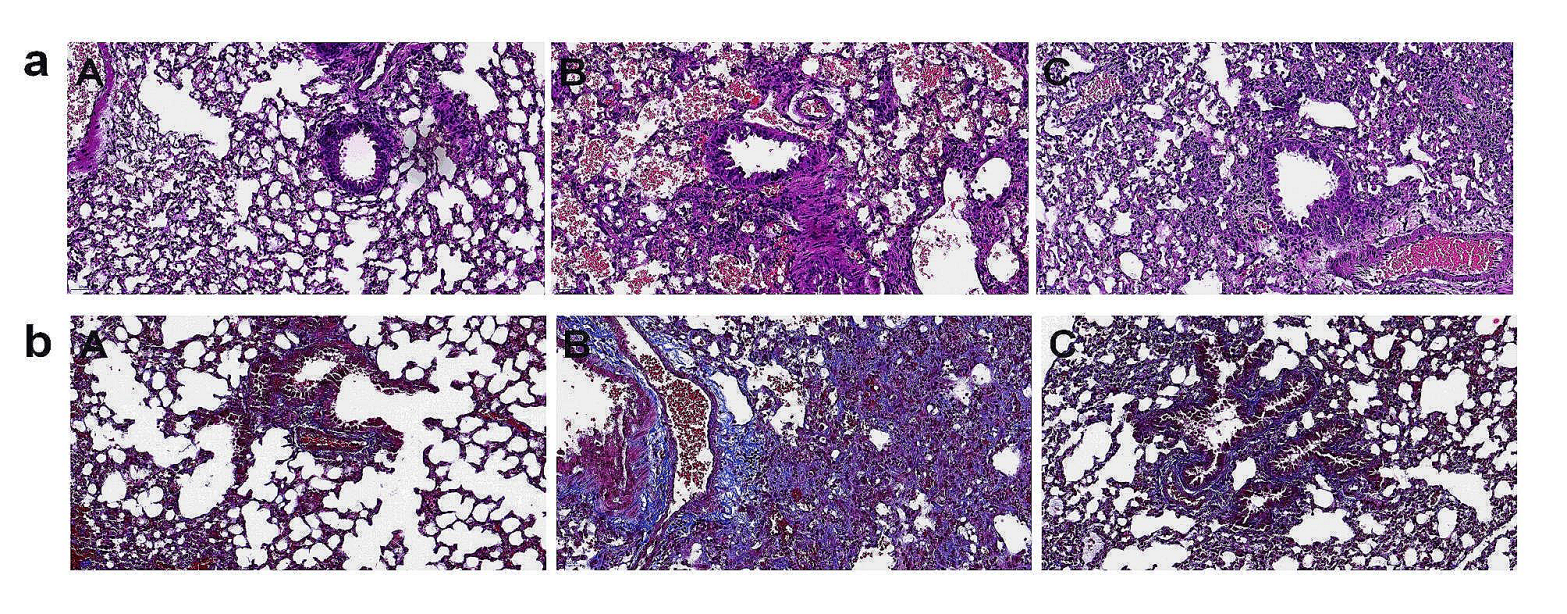
The effect of YPG on bronchial asthma in rats. Sections were stained with H&E (**a**). Sections were stained with Masson’s trichrome (**b**). Magnification, ×400; scale bars, 50 μm. Control group (A), Model group (B), YPD group (C)

In the control group, there was no infiltration of inflammatory cells to the bronchus, the bronchial wall was smooth, cells were arranged in a regular manner, and the thickness of the soft muscle layer was normal. Compared with the control group, there were many eosinophils, monocytes, and lymphocytes around the bronchial wall in the asthma group. Proliferation of airway epithelial cells, thickening of the smooth-muscle layer, as well as thickening and prolongation of the bronchial mucosa were also documented (Fig. 1a). After YPD treatment, the infiltration of inflammatory cells (eosinophils and lymphocytes) in rats with asthma was reduced significantly, and the thickness of the bronchial wall and smooth-muscle layer was close to that of the control group.

Staining (Masson’s trichrome) showed the structure of lung tissue in the control group to be normal with few collagen fibers (blue) or muscle fibers (red) [20] (Fig. 1b). Compared with the control group, the bronchial wall and alveolar wall were obviously thickened and consolidated in the model group, with a significant increase in the areas stained blue and red. After YPD treatment, the number of collagen fibers and muscle fibers was reduced significantly.

### Identification of DEGs

**Fig. 2:**
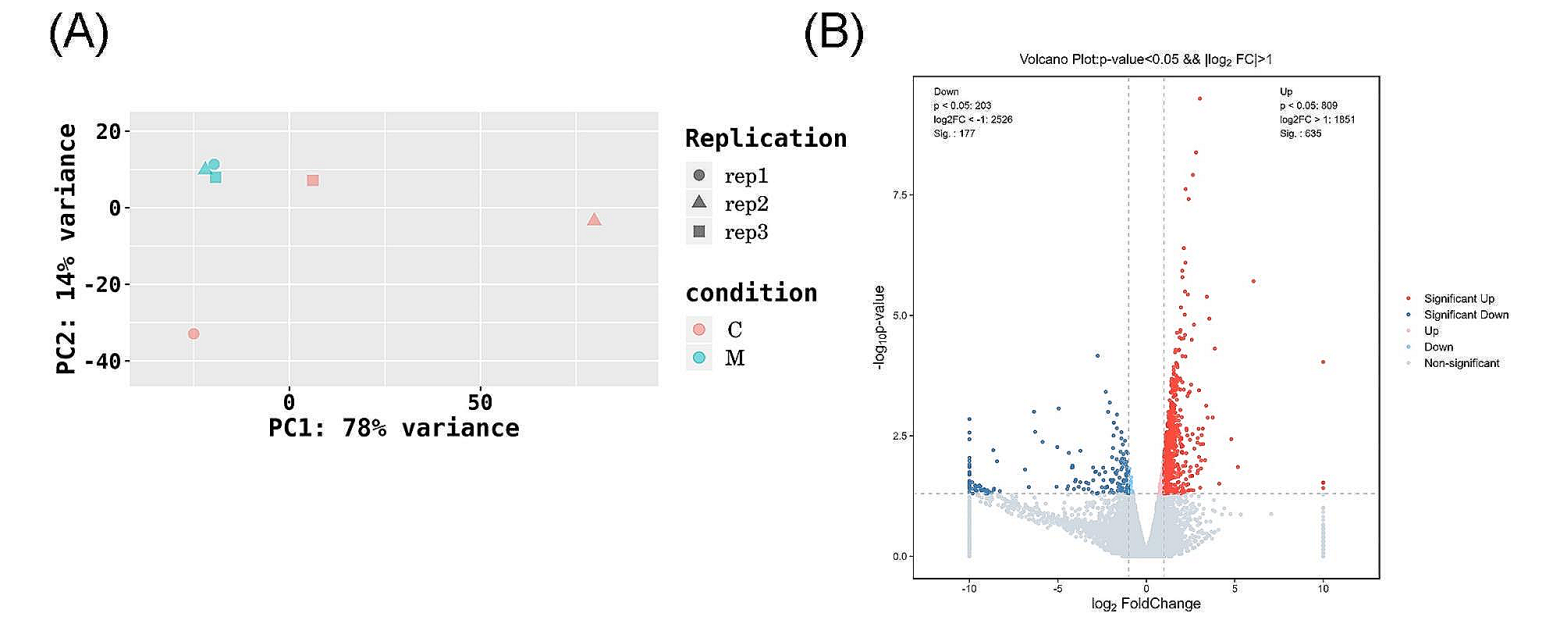
(**A**) PCA of gene expression between samples. (**B**) Volcano plot of DEGs in control and model groups. C: Control group, M: Model group

RNA-sequencing was used to understand multifaceted mechanisms in rats with asthma [26]. We measured mRNA expression of each sample in control and asthma groups. The results of principal component analysis showed considerable separation between these two groups, indicating that the transcriptome sequencing was of high quality and could be analyzed further (Fig. 2A). A total of 812 DEGs were identified in the model group compared with the control group (Fig. 2B) (635 genes with upregulated expression and 177 genes with downregulated expression).

### Analysis of DEGs with altered function after asthma onset

Classification of gene function can describe the properties of genes and products comprehensively, and such classification is updated constantly. Classification is based mainly on biological process (BP), cellular component (CC), and molecular function (MF). Comparison of data from analyses of functional enrichment with the genomic background aids understanding of which biological functions are associated significantly with DEGs. DEGs had to meet the criteria of corrected p-value (Q-value) ≤ 0.05. The biological functions of DEGs that met these criteria were determined.

We performed functional-enrichment analysis on DEGs with upregulated expression and downregulated expression in the asthma group: 635 genes had upregulated expression and 6230 GO terms were identified (4849 for BP, 528 for CC, and 853 for MF). The top-five GO terms with the smallest p-value (i.e., the most significant enrichment) were selected for each GO classification.

**Fig. 3:**
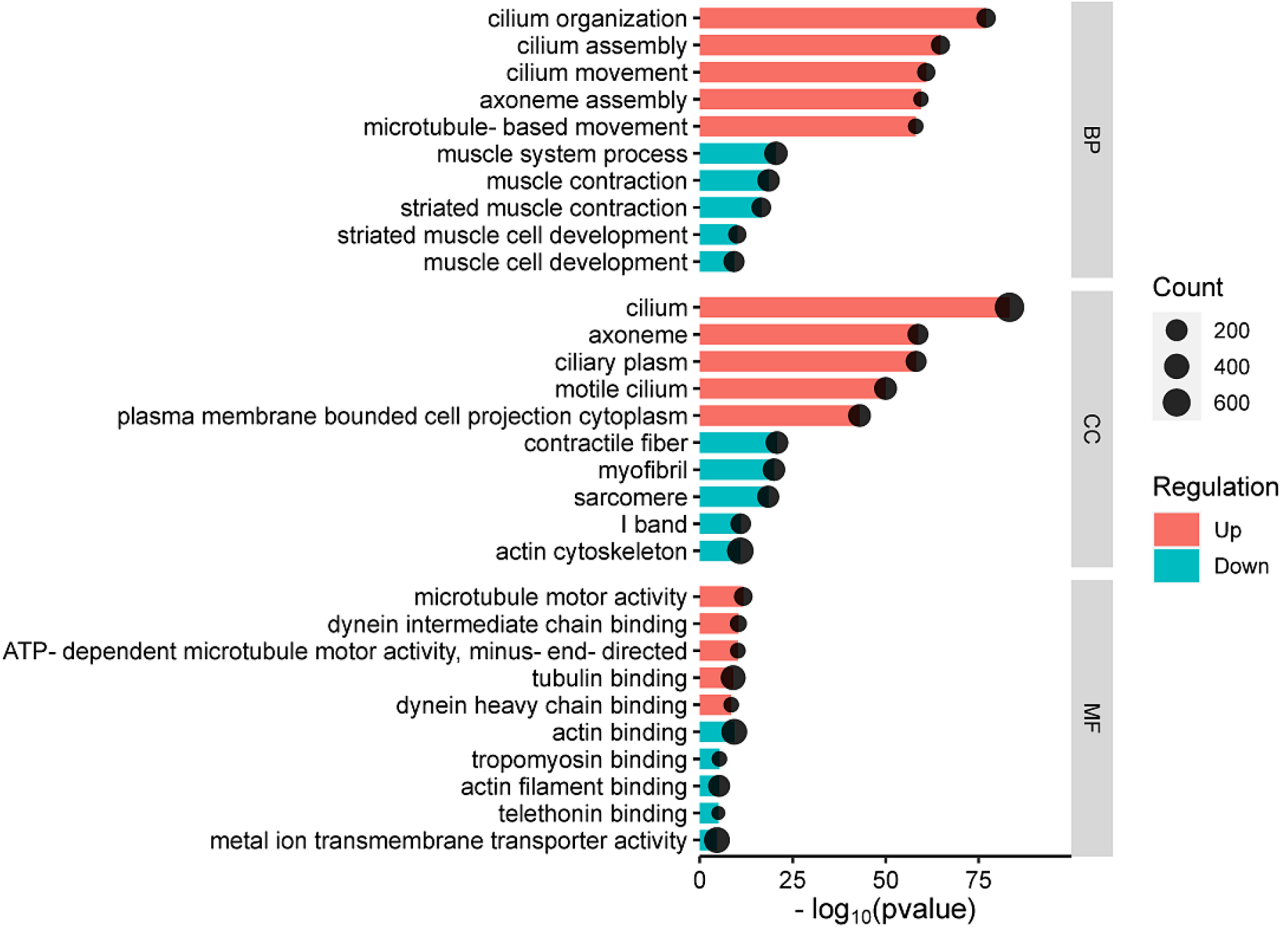
Analysis of signaling-pathway enrichment of DEGs between the control group compared with the model group

The top-five enriched functions for BP were ‘cilium organization’ (GO: 0044782), ‘cilium assembly’ (GO: 0060271), ‘cilium movement’ (GO: 0003341), ‘axoneme assembly’ (GO: 0035082), and ‘microtubule-based movement’ (GO: 0007018) (Fig. 3). The top-five enriched functions for CC were ‘cilium’ (GO: 0005929), ‘axoneme’ (GO: 0005930), ‘ciliary plasm‘ (GO: 0097014), ‘motile cilium’ (GO: 0031514), and ‘plasma membrane bounded cell projection cytoplasm’ (GO: 0032838). The top-five enriched functions for MF were ‘microtubule motor activity’ (GO: 0003777), ‘dynein intermediate chain binding’ (GO: 0045505), ‘ATP-dependent microtubule motor activity, minus-end-directed’ (GO: 0008569), ‘tubulin binding’ (GO: 0015631), and ‘dynein heavy chain binding’ (GO: 0045504).

The 177 genes with downregulated expression were enriched with 3788 GO terms (2919 for BP, 346 for CC, and 523 for MF). The top-five enriched functions for BP were ‘muscle system process’ (GO: 0003012), ‘muscle contraction’ (GO: 0006936), ‘striated muscle contraction’ (GO: 0006941), ‘striated muscle cell development’ (GO: 0055002), and ‘muscle cell development’ (GO: 0055001). The top-five enriched functions for CC were ‘contractile fiber’ (GO: 0043292), ‘myofibril’ (GO: 0030016), ‘sarcomere’ (GO: 0030017), ‘I band’ (GO: 0031674), and ‘actin cytoskeleton’ (GO: 0015629). The top-five enriched functions for MF were ’actin binding’ (GO: 0003779), ‘tropomyosin binding’ (GO: 0005523), ‘actin filament binding’ (GO: 0051015), ‘telethonin binding’ (GO: 0031433), and ‘metal ion transmembrane transporter activity’ (GO: 0046873).

### Analysis of signaling pathways of DEGS altered after asthma onset

**Fig. 4:**
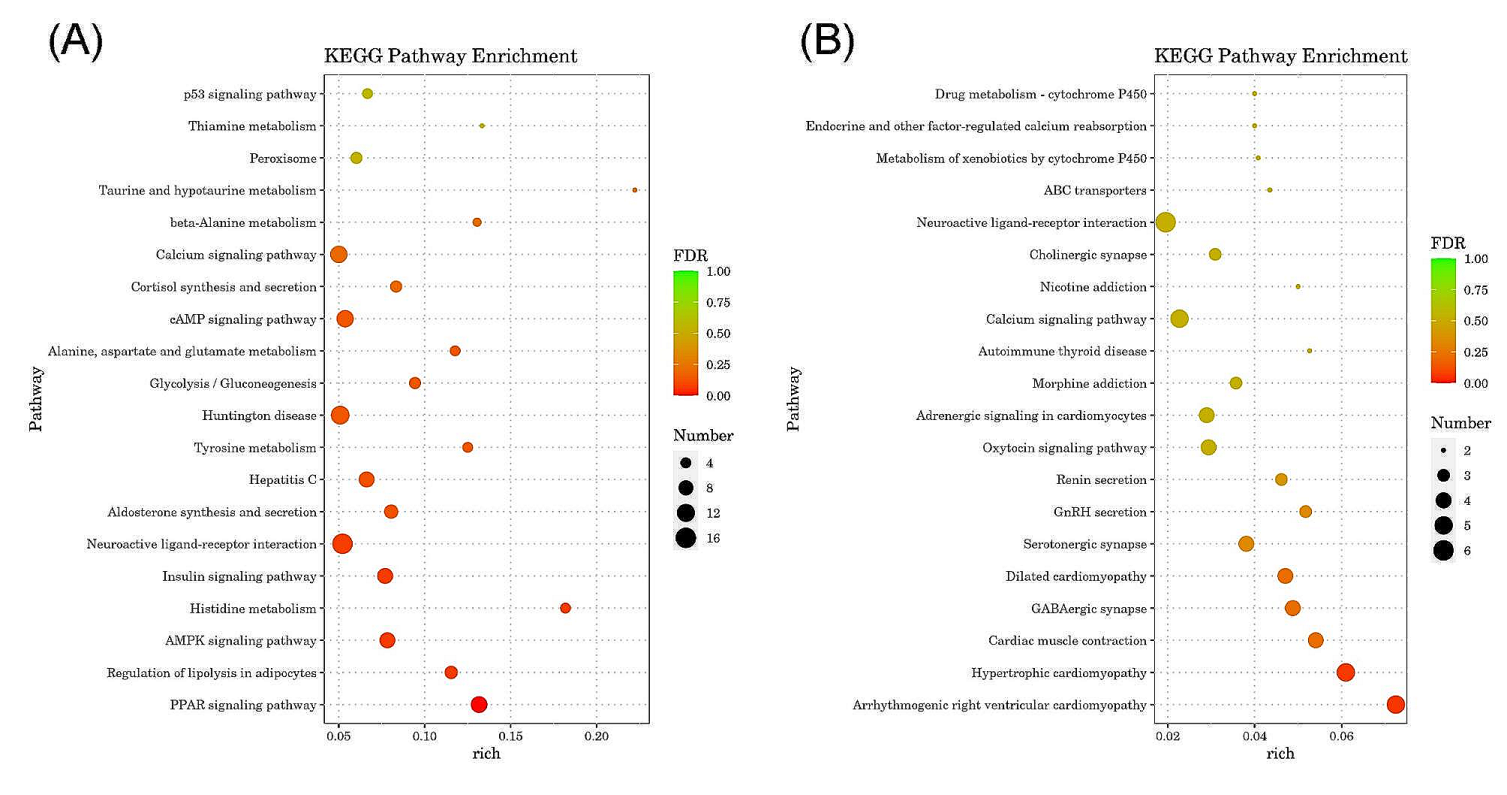
KEGG pathway enrichment analysis of DEGs between the control group compared to the model group

Analysis of signaling-pathway enrichment was done using the KEGG database. We discovered that 635 upregulated genes were enriched in 226 signaling pathways. The 10 most significantly enriched signaling pathways were ‘PPAR signaling pathway’ (FDR: 0.002001573), ‘regulation of lipolysis in adipocytes’ (FDR: 0.069244813), ‘AMPK signaling pathway’ (FDR: 0.069244813), ‘histidine metabolism’ (FDR: 0.069244813), ‘insulin signaling pathway’ (FDR: 0.069244813), ‘neuroactive ligand–receptor interaction’ (FDR: 0.074370072), ‘aldosterone synthesis and secretion’ (FDR: 0.120696471), ‘hepatitis C’ (FDR: 0.120696471), ‘tyrosine metabolism’ (FDR: 0.139915558), and ‘Huntington disease’ (FDR: 0.139915558). The smaller the FDR value, the more significant was the enrichment. We selected the top-20 signaling pathways with the smallest FDR value (Fig. 4A). The 177 genes with downregulated expression were enriched in 151 signaling pathway. The 10 most significantly enriched signaling pathways were ‘arrhythmogenic right ventricular cardiomyopathy’ (FDR: 0.057189133), ‘hypertrophic cardiomyopathy’ (FDR: 0.063430675), ‘cardiac muscle contraction’ (FDR: 0.21866133), ‘GABAergic synapse’ (FDR: 0.21866133), ‘dilated cardiomyopathy’ (FDR: 0.21866133), ‘serotonergic synapse’ (FDR: 0.334112021), ‘GnRH secretion’ (FDR: 0.334112021), ‘renin secretion’ (FDR: 0.395775367), ‘oxytocin signaling pathway’ (FDR: 0.525036286), and ‘adrenergic signaling in cardiomyocytes’ (FDR: 0.525036286). Based on the FDR value, we selected the top-20 most significantly enriched signaling pathways (Fig. 4B).

### PPI network

**Fig. 5:**
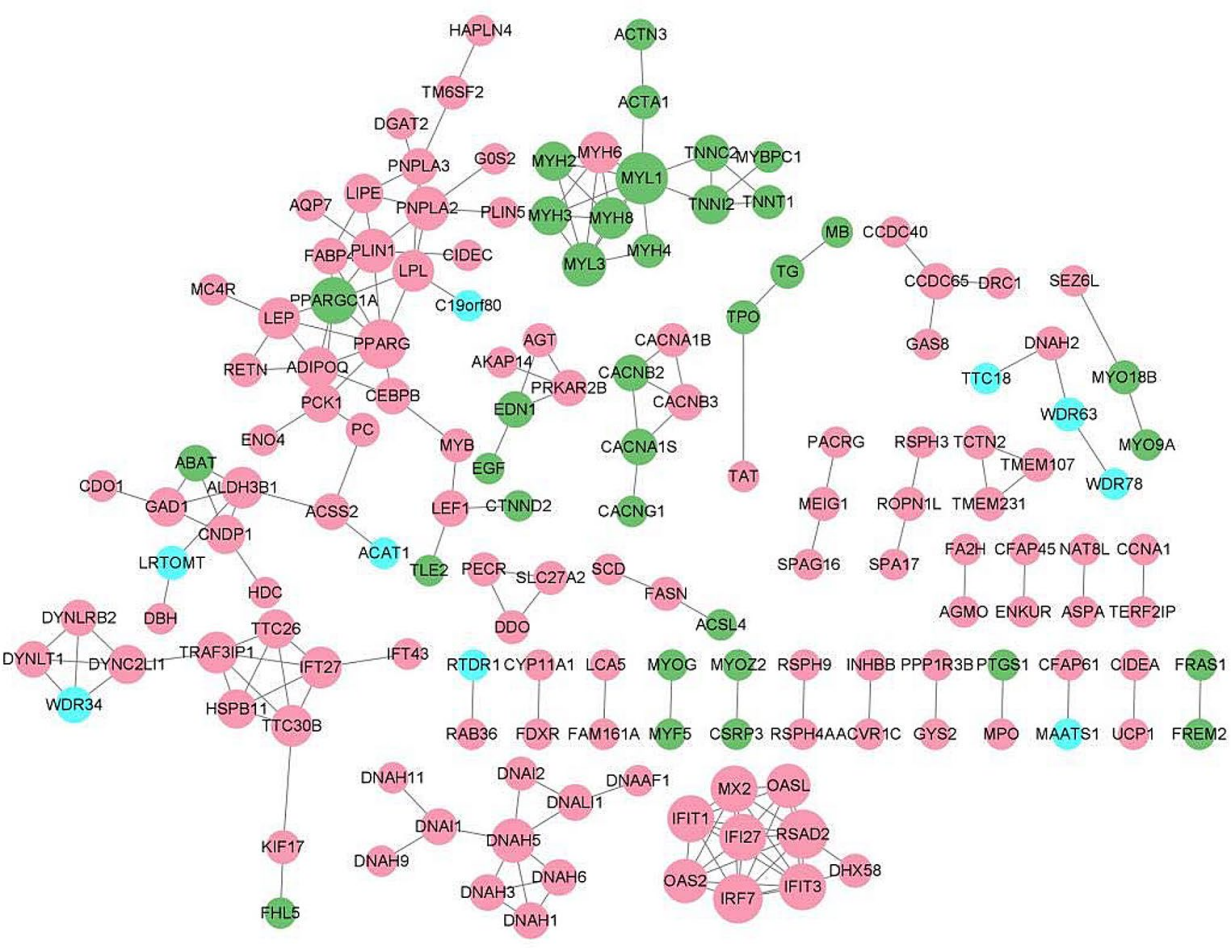
Protein–protein interaction (PPI) network of DEGs in control and model groups. Red nodes indicate gene with upregulated expression and green nodes indicate genes with downregulated expression. Blue nodes indicates genes with unchanged expression

Genes with significant differential expression were used to construct a PPI network based on the STRING database. The PPI network had 154 nodes and 189 edges (Fig. 5), among which the higher ranked degree values were recombinant myosin light chain 1 (MYL1), radical S-adenosyl methionine domain-containing protein 2 (RSAD2), interferon regulatory factor 7 (IRF7), and peroxisome proliferative activated receptor G (PPARG).

**Fig. 6:**
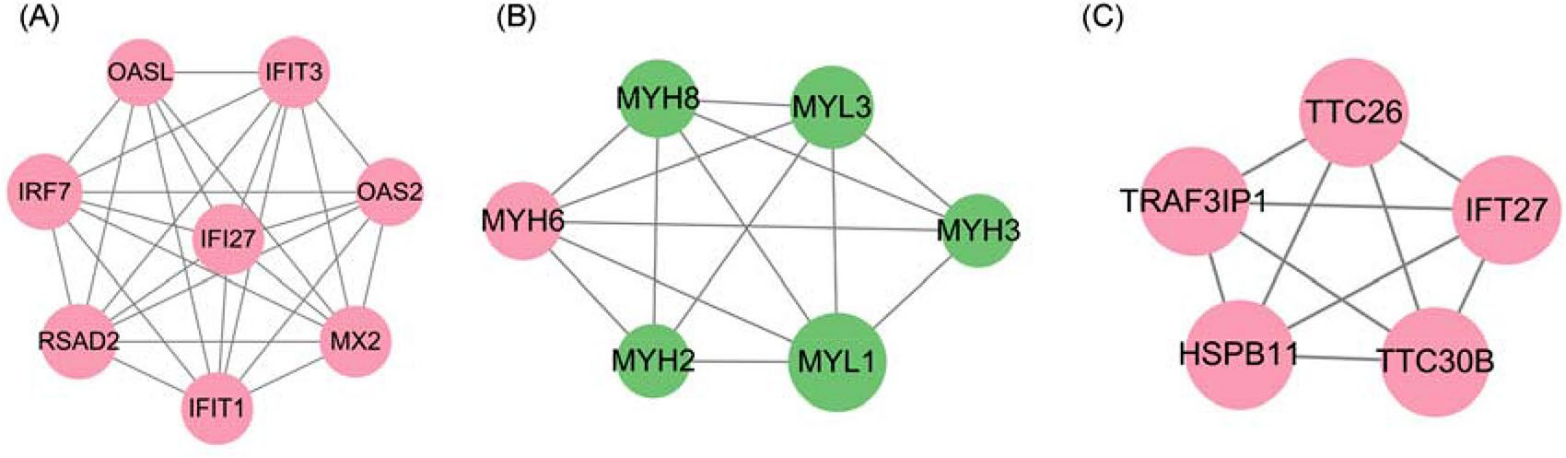
(**A**–**C**) PPI network of three key modules. Red nodes indicate genes with upregulated expression and green nodes indicate genes with downregulated expression

The top-five DEGs with upregulated expression and downregulated expression are listed in Table 3. We used the MCODE plugin in Cytoscape (https://cytoscape.org/) [27] to analyze the 12 modules obtained. Modules with a score > 5 and > 5 nodes were filtered and visualized according to ranking of the module score (Fig. 6). Most of DEGs belonging to the three modules had high expression in the asthma group.

**Table 3:** Top 5 up-regulated and down-regulated genes

Gene_id	Gene_name	p-value	Up/down	Degree
ENSRNOG00000007539	RSAD2	0.016825	up	8
ENSRNOG00000017414	IRF7	0.017256	up	8
ENSRNOG00000008839	PPARG	0.000188	up	8
ENSRNOG00000036603	IFIT1	1.000000	up	7
ENSRNOG00000001963	MX2	0.009853	up	7
ENSRNOG00000013262	MYL1	0.047501	down	9
ENSRNOG00000004473	PPARGC1A	0.019420	down	7
ENSRNOG00000020955	MYL3	0.039811	down	6
ENSRNOG00000068010	MYH8	0.033754	down	5
ENSRNOG00000046276	MYH3	0.027834	down	4

### RT-qPCR verification of DEGs

**Fig. 7:**
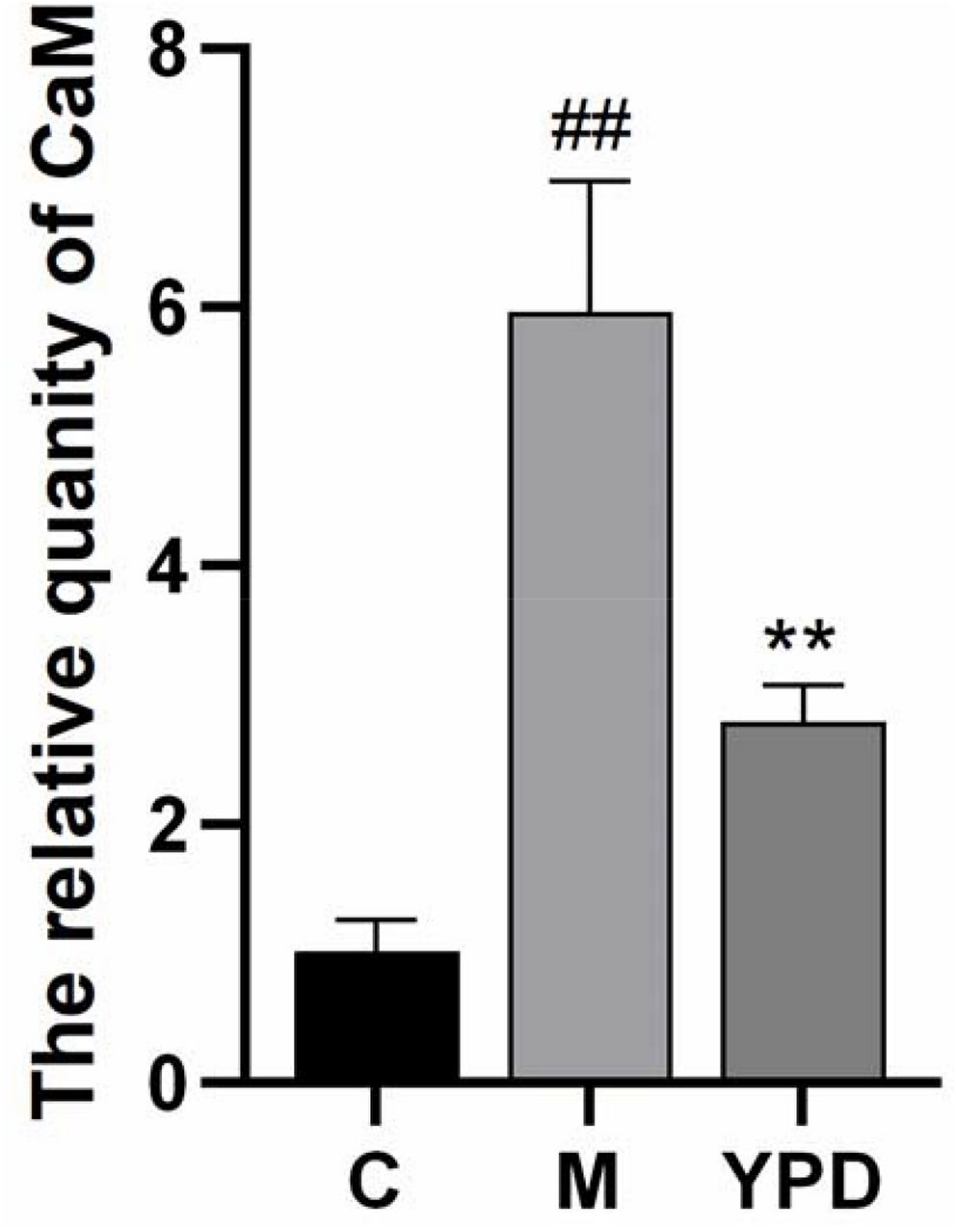
Effect of YPD on the Ang-II/PLA/CaM signaling axis detected by RT-qPCR. C: Control group; M: Model group; YPD: YPD group. P^##^ <0.01, P^**^ <0.01

Differential expression of genes was detected by RT-qPCR (Fig. 7), Expression of Ang-II, PLA and CaM in the asthma group was increased significantly compared with that in the control group (p < 0.05). After intervention with YPD, expression of Ang-II, PLA and CaM in the YPD group was downregulated significantly compared with that in the asthma group (p < 0.05). These results suggested that YPD may inhibit the Ang-II/PLA/CaM signaling axis.

### Effect of YPD on the intracellular concentration of Ca^2+^

**Fig. 8:**
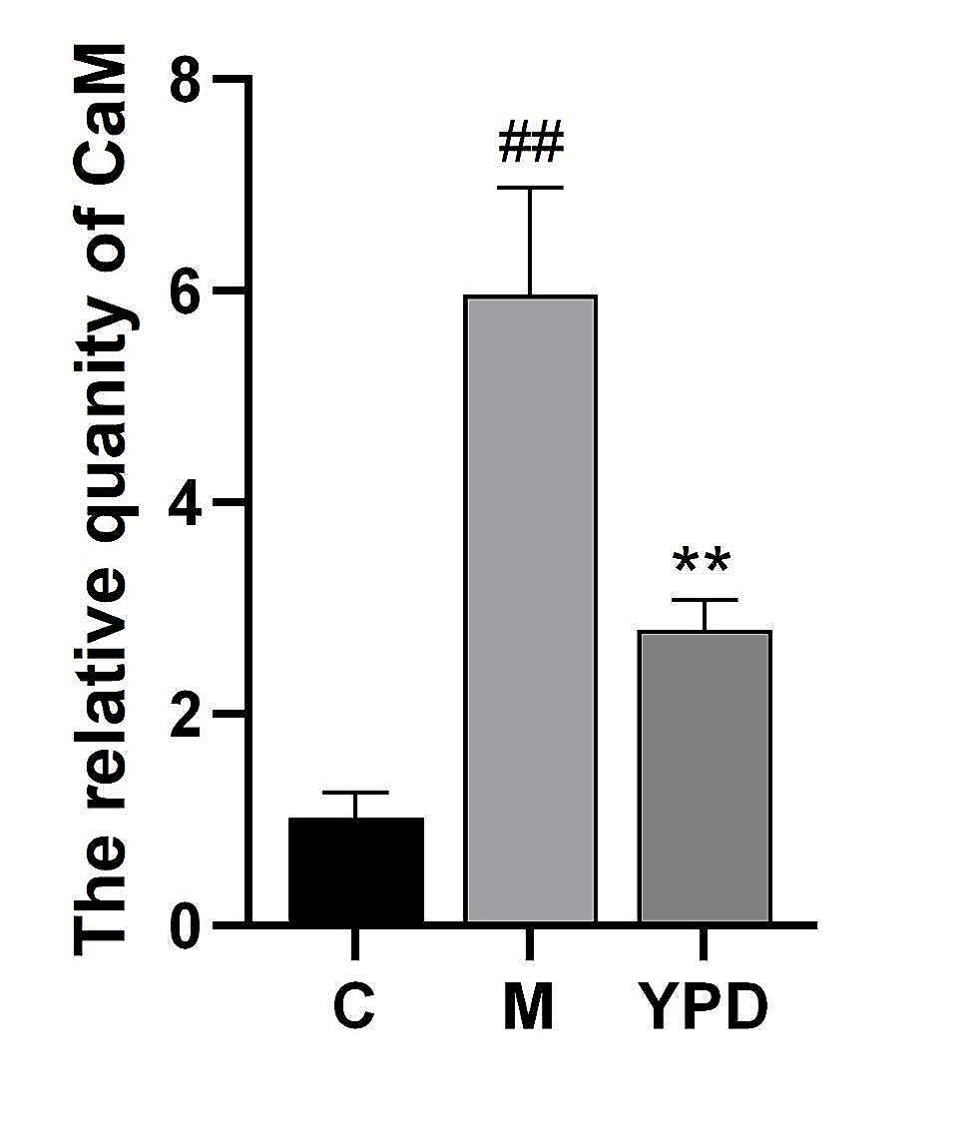
Effect of YPD on the Ca^2+^ concentration. C: Control group; M: Model group; YPD: YPD group. P^##^ <0.01, P^**^ <0.01

A calcium detection kit based on use of methylthymol blue in microplates was used to measure the intracellular concentration of Ca^2+^. Compared with the control group, the Ca^2+^ concentration in the asthma group was increased significantly (p < 0.01) (Fig. 8). After YPD intervention, the Ca^2+^ concentration in the YPD group was downregulated significantly compared with that in the asthma group. These results suggested that YPD may reduce the intracellular Ca^2+^ concentration in rats suffering from asthma.

## Discussion

YPD have been shown to be effective in controlling asthma, including inhibiting excessive proliferation of bronchial cells and alleviating inflammation [17, 18]. However, due to the multiple-component nature of TCM formulations, how YPD controls asthma in rats is not known.

We explored how YPD protects against asthma using RNA-sequencing. RNA-sequencing in the asthma group and control group revealed 812 DEGs, of which 635 genes had upregulated expression and 177 genes had downregulated expression, compared with the control group. Analyses of functional enrichment (using the GO database), signaling-pathway enrichment (using the KEGG database), and PPI networks provided deeper understanding of the pathologic mechanisms of asthma. We hypothesized that YPD may control asthma pathogenesis by binding Ca^2+^, inorganic cation transmembrane transporter activity, and microtubule motor activity. We also observed some canonical signaling pathways (e.g., peroxisome proliferator-activated receptor, calcium, cyclic adenosine monophosphate).

Asthma is induced by inflammation, remodeling, and hyperresponsiveness of the airways [21, 22]. Airway hyperreactivity includes intense constriction of the airways and airway spasm. Airway smooth muscle (ASM) cells are the main cell type that cause bronchial hyperresponsiveness [23], so they are also considered to be a target to combat bronchiectasis. In normal airways, ASM cells contract to regulate the airway diameter and bronchial motor tension. In asthma, there is a complex relationship between ASM cells and inflammatory cells (e.g., mast cells, T lymphocytes). Airway contraction is caused mainly by ASM contraction. There are many reasons for ASM contraction, but recent studies have shown that ASM contraction may be related to an increased level of Ca^2+^ [24, 25].

**Fig. 9:**
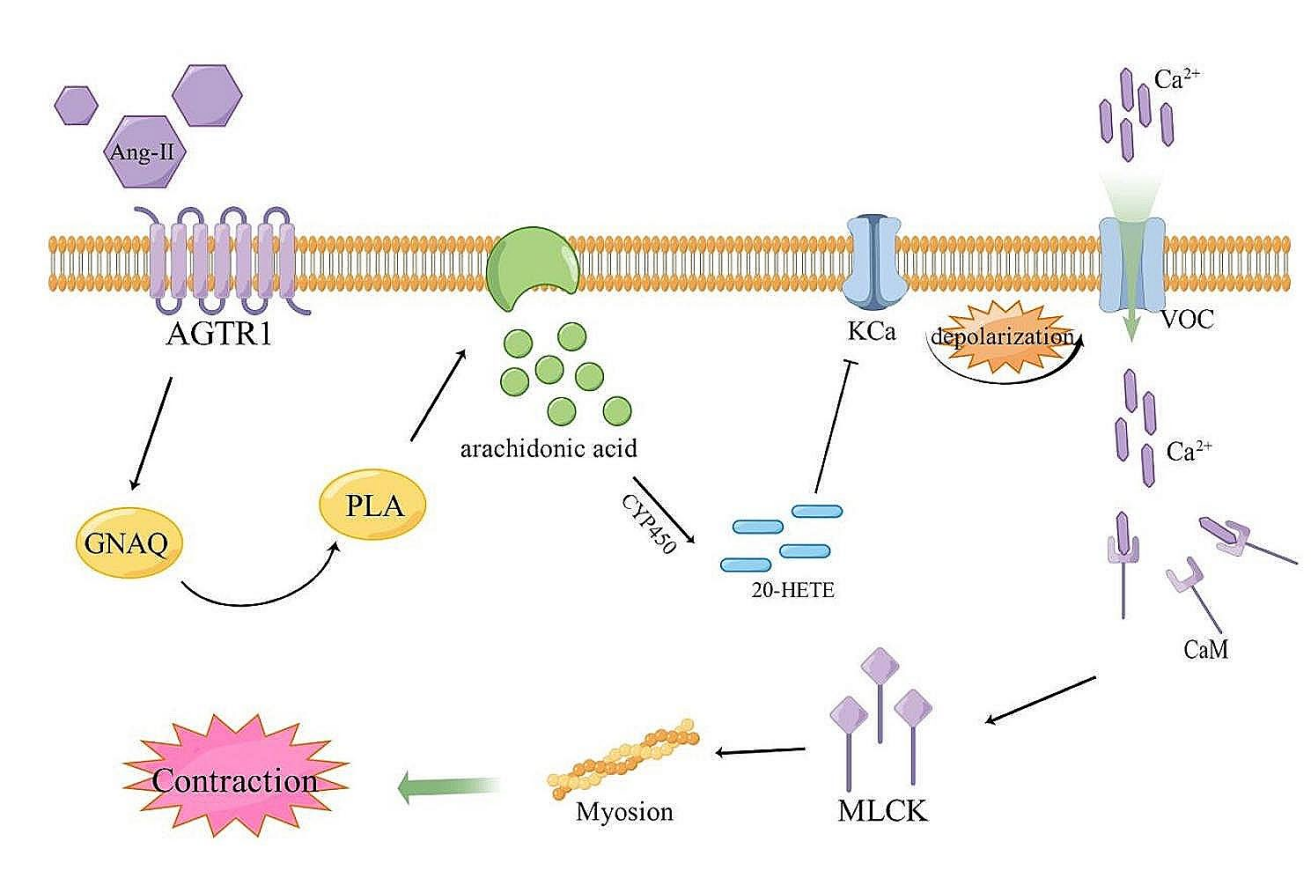
Ang-II/PLA/CaM signaling axis in vitro

The current strategy to control an acute attack of asthma is to relax ASM and dilate the bronchus [26]. Ca^2+^ regulation is crucial to the contraction and relaxation of ASM [27]. If the Ca^2+^ concentration in ASM cells increases, Ca^2+^ binds to calmodulin and activates myosin light chain kinase (MLCK), resulting in MLC phosphorylation [28]. Phosphorylation of MLC increases the activity of myosin adenosine triphosphate (ATP)ase, which hydrolyzes ATP to release energy [29]. Actin-myosin is induced to transbridge circulation, resulting in shortened ASM. Therefore, regulation of the Ca^2+^ concentration in ASM cells is essential for controlling asthma symptoms. The intracellular Ca^2+^ concentration is regulated by calcium channels [30], ryanodine receptors [31], calcium pumps [32], and Na^+^/Ca^2+^ transporters [33], which can mediate the transmembrane flow of Ca^2+^. Ca^2+^-channel transport is one of the main ways in which Ca^2+^ enters cells. Ca^2^ channels in cell membranes can be divided into five categories (voltage-operated, voltage-dependent, receptor-operated, second messenger-operated, and mechanically operated). Among them, voltage-dependent Ca^2^ channels are the most important because they are ubiquitous in various tissues in the body, and are the main pathway of Ca^2+^ influx [34]. If a cell is depolarized, voltage-operated Ca^2+^ channels transfer Ca^2+^ from outside to inside the cell. Studies have shown that 20-hydroxyeicosatetraenoic acid (20-HETE) can cause the depolarization of vascular smooth muscle cells [35]. 20-HETE is an important metabolite in the cytochrome P450 metabolic pathway of arachidonic acid. The latter is stored in the cell membrane and released into the cytoplasm through regulation of phospholipase A [36]. Angiotensin can increase the PLA level through a series of reactions. Therefore, the angiotensin-2 signaling axis may have an important regulatory role in ASM contraction (Fig. 9, by Figdraw).

Analysis of signaling-pathway enrichment revealed five DEGs to be enriched with regard to contraction of vascular smooth muscle, among which expression of Ang-II, PLA and CaM was increased in the asthma group, and YPD could reverse this increase. Functional-enrichment analysis showed that YPD-based control of asthma pathogenesis may be related to Ca^2+^ binding and inorganic cation transmembrane transporter activity. Therefore, we measured expression of the Ang-II/PLA/CaM signaling axis in asthma and YPD treatment to ascertain if ASM is related to Ca^2+^ (the Ca^2+^ level in the all three groups was measured simultaneously). The Ang-II/PLA/CaM signaling axis was activated in the asthma group and the Ca^2+^ level increased; YPD could inhibit the Ang-II/PLA/CaM signaling axis and reduce the Ca^2+^ concentration.

## Conclusions

We used deep RNA-sequencing to reveal that YPD could reduce the Ca^2+^ concentration by the Ang-II/PLA/CaM signaling axis in vitro. Our study provides insights into the potential applicability of YPD in asthma therapy.

## Data Availability

The datasets used and/or analysed during the current study areavailable from the GEO database ((http://www.ncbi.nlm.nih.gov/geo) [Accession number: GSE247032 (http://www.ncbi.nlm.nih.gov/geo/query/acc.cgi?acc=GSE247032)]. The original data are available from the corresponding author upon reasonable request.

## Abbreviations

YPD: Yanghe Pingchuan decoction
DEGs: differentially expressed genes
TCM: Traditional Chinese medicine
FDR: false discovery rate
GO: Gene Ontology
KEGG: Kyoto Encyclopedia of Genes and Genomes
PPI: protein–protein interaction
RT-qPCR: Real-time reverse transcription-quantitative polymerase chain reaction
ASM: Airway smooth muscle
